# Characterization of the microbial communities in wheat tissues and rhizosphere soil caused by dwarf bunt of wheat

**DOI:** 10.1038/s41598-021-85281-8

**Published:** 2021-03-11

**Authors:** Tongshuo Xu, Wenli Jiang, Dandan Qin, Taiguo Liu, Jianmin Zhang, Wanquan Chen, Li Gao

**Affiliations:** 1 State Key Laboratory for Biology of Plant Disease and Insect Pests, Institute of Plant Protection, Chinese Academy of Agricultural Sciences, Beijing, China; 2 School of Agriculture, Yangtze University, Jingzhou, China

**Keywords:** Microbiology, Fungi, Pathogens

## Abstract

Dwarf bunt of wheat, which is caused by *Tilletia controversa* J.G. Kühn, is a soil-borne disease which may lead up to an 80% loss of yield together with degradation of the quality of the wheat flour by production of a fishy smell. In this study, high-throughput sequencing technology was employed to characterize the microbial composition of wheat tissues (roots, spikes, first stem under the ear, and stem base) and rhizosphere soil of wheat varieties that are resistant and susceptible to *T. controversa*. We observed that the soil fungal community abundance and diversity were higher in resistant varieties than in susceptible varieties in both inoculated and uninoculated wheat, and the abundances of Sordariomycetes and Mortierellomycetes increased in the resistant varieties infected with *T. controversa*, while the abundances of Dothideomycetes and Bacteroidia increased in the susceptible varieties. Regarding the bacteria present in wheat tissues, the abundances of Chloroflexi, Bacteroidetes, Gemmatimonadetes, Verrucomicrobia and Acidobacteria in the ear and the first stem under the ear were higher than those in other tissues. Our results indicated that the abundances of Sordariomycetes, Mortierellomycetes, Leotiomycetes, *Chryseobacterium* and *Massilia* were higher in *T. controversa*-infected resistant varieties than in their controls, that Dothideomycetes, Bacteroidia, *Nocardioides* and *Pseudomonas* showed higher abundances in *T. controversa*-infected susceptible varieties, and that *Curtobacterium*, *Exiguobacterium*, *Planococcus*, and *Pantoea* may have higher abundances in both *T. controversa-*infected susceptible and resistant varieties than in their own controls.

## Introduction

Wheat (*Triticum aestivum* L.) is an important food crop that humans have been consuming for 5000 years^1^. The quality and yield of wheat are affected by various factors^2^. Dwarf bunt in wheat, which is caused by *Tilletia controversa* J.G. Kühn, spreads through seeds or soil^3^ and is a disease of quarantine significance in many countries^4^. This disease often harms winter wheat in areas covered by snow for long periods in winter. Wheat Dwarf bunt can lead to a reduction in wheat production, degrade flour quality and produce a rotten fish odour. Resistant varieties of wheat contribute considerably to controlling this disease^5^. The teliospores of *T. controversa* have strong resistance to stress and can survive for 10 years under favourable environments^6^.

Plant microbiota can help to maintain the health of plants and can provide important genetic variability, which is of strong significance for plant resistance to biotic and abiotic stress^7^. Plant entophytic bacteria are parasitic bacteria that are widely observed in the tissues of plants in nature^8^. These bacteria can exist in various parts of the plant, including the aboveground, belowground, and seed parts^9^, and they can promote plant growth by fixing nitrogen, producing plant hormones, and improving drought resistance^10–12^; also, these bacteria can protect host plants from damage by inhibiting the growth of pathogenic bacteria^13–15^. In a recent study on the response of wheat endophytes to stripe rust, it was observed that the abundance of endophytes in roots was higher than that in stems and leaves, and the abundances of endophytes in resistant and susceptible varieties was observed to vary considerably^16^.

Rhizosphere soil serves as a bridge between microbes and plant roots. This soil enables materials and energy to be exchanged between plants and microbes. The rhizosphere microbiota promotes plant growth and health by enhancing plant resistance to adverse conditions or improving plant nutrient absorption^17–19^. The rhizosphere bacterial community in the soil can strongly reduce the morbidity and mortality in tobacco caused by mixed *Fusarium-Alternaria* disease^20^. Thilagam and Hemalatha^21^ found that plant growth-promoting rhizobacterial (PGPR) actinobacterial isolates can effectively suppress chili anthracnose. At the same time, soil microbes can also increase the resistance of plants to some microbial stresses by enhancing plant drought resistance by intercepting hormones in plants^22^. The rhizosphere microbiota can also affect the nutritional status of plants. The symbiotic relationship between legumes and nitrogen-fixing *rhizobia* is a typical example of how soil microbiota helps plants absorb nitrogen^23^. Some rhizosphere microbiota can promote iron and phosphorus absorption by plants through mineralization, dissolution, or the secretion of iron carriers^19, 24^. Similarly, plants can also affect the structure and composition of the rhizosphere microbial community. Resistant and susceptible varieties exhibit differences between their rhizosphere microbial communities. In a study of the resistance and susceptibility of watermelon to *Fusarium oxysporum* f. sp. *niveum*, An et al.^25^ observed that the populations of actinomycetes in the rhizosphere soil of resistant varieties are more abundant than those of susceptible varieties, but the fungal community exhibits the opposite property. Sun et al.^26^ compared the difference in the microbial diversity of rhizosphere soil between resistant and susceptible banana varieties and observed that the diversity of the resistant microbiota was higher than that of the susceptible microbiota. In this study, based on an analysis of the 16S rRNA and ITS genomic regions of bacterial and fungal samples, we explored microbial communities in tissue samples from wheat varieties that are resistant or sensitive to *T. controversa* infection, as well as in rhizosphere soil, as these microbial communities may contribute to the control of dwarf bunt in wheat.

## Results

### Detection of *T. controversa* in wheat leaves

Based on the DNA from the leaves of the inoculated plants and control plants of the four varieties, successful infection by *T. foetida* was confirmed by a specific band (372 bp) from inoculated leaf samples (Supplementary Fig. S1).

### Sequencing data statistics and evaluation

Through Illumina MiSeq sequencing, 1,709,173 optimized fungal sequences were obtained from 24 rhizosphere soil samples, and 1,532,304 effective sequences were obtained; a total of 1,944,678 optimized bacterial sequences were obtained from 84 samples, including 24 rhizosphere soil samples, 24 root samples, 12 spike samples, 12 stem base samples and 12 first stem under the ear samples, and 475,524 effective sequences were obtained. The dilution curve according to the Shannon index reached a plateau (Supplementary Fig. S2), indicating that the quantity of sequencing data was sufficient for subsequent experiments. According to the similarity level, all sequences were divided into OTUs, and sequences with 97% or greater similarity to OTU representative sequences were selected for statistical analysis of biological information. A total of 1721 fungal OTUs and 3824 bacterial OTUs were obtained, and species classification analysis was performed (Supplementary Table S1).

### Microbial diversity of rhizosphere soil

A comparison of richness and diversity indexes, which are the average of three samples, demonstrated the differences in microorganisms among soil samples. For the fungal community (Supplementary Table S2), the Shannon index indicated that the diversity of the resistant varieties Mianyang 26/Yumai 47 (CK26, 3.77; R26, 4.47) and Yumai 49 * 4/Lankao dwarf 8 (CK49, 3.53; R49, 4.33) was higher in *T. controversa*-inoculated wheat than in uninoculated wheat, and the diversity of A-45 (CK45, 3.43; S45, 3.56) was also higher in inoculated wheat; however, the diversity of A-44 (CK44, 2.64; S44, 2.62) decreased slightly. Regardless of whether inoculated wheat or uninoculated wheat was used, the diversity of resistant varieties was higher than that of susceptible varieties. In terms of species richness, the Ace and Chao indexes showed that the richness of the infected wheat Yumai 49 * 4/Lankao dwarf 8 (Ace: CK49, 820.46, R49, 899.84; Chao, CK49, 793.82, R49, 899.41) and A-45 (Ace: CK45, 344.68, S45, 769.54; Chao: CK45, 342.11, S45, 762.84) increased compared with the control, while the abundance of A-44 (Ace: CK44, 391.57, S44, 335.47; Chao: CK44, 364.00, S44, 323.75) was observed to decrease. The trends of the Ace and Chao index in Mianyang 26/Yumai 47 (Ace: CK26, 933.55, R26, 912.39; Chao: CK26, 854.66, R26, 901.53) were inconsistent. The fungal diversity of the resistant varieties inoculated with *T. controversa* was higher than that of uninoculated wheat, while the fungal communities of the susceptible varieties were notably diverse. We also found that among the uninoculated wheat varieties, the richness of fungi in the samples of Mianyang 26/Yumai 47 (Ace: 933.55; Chao: 854.66) and Yumai 49 * 4/Lankao dwarf 8 (Ace: 820.46; Chao: 793.82) were relatively high. In wheat inoculated with *T. controversa*, the richness of fungi in the samples of Mianyang 26/Yumai 47 and Yumai 49 * 4/Lankao dwarf 8 was also higher than that in the two susceptible varieties. These results indicate that regardless of whether plants were inoculated with *T. controversa*, the diversity and richness of the fungal community of the resistant varieties was higher than that of the susceptible varieties.

In Supplementary Table S3, studies of the diversity of bacterial communities in the soil showed that the bacterial diversity and richness of Mianyang 26/Yumai 47 (Shannon: CK26, 6.03, R26, 6.09; Ace: CK26, 1447.84, R26, 1563.08; Chao: CK26, 1346.46, R26, 1374.44) and A-45 (Shannon: CK45, 5.03, S45, 6.06; Ace: CK45, 976.36, S45, 1311.98; Chao: CK45, 868.62, S45, 1258.17) increased compared with those of uninoculated wheat. However, the diversity of Yumai 49 * 4/Lankao 8 (Shannon: CK49, 6.06, R49, 6.06; Ace: CK49, 1460.83, R49, 1335.21; Chao: CK49, 1362.93, R49, 1294.33) was almost unchanged, and the richness was observed to decrease. Also, the bacterial diversity and abundance in soil from A-44 decreased (Shannon: CK44, 4.25, S44, 2.63; Ace: CK44, 794.30, S44, 393.43; Chao: CK44, 703.28, S44, 305.51).

### Microbial diversity of wheat tissue

We also studied the endophyte community in different parts of wheat plants, including the roots, ears, first stem under the ear and base of the stem, from the resistant variety Mianyang 26/Yumai 47 and the susceptible variety A-45. For the microorganisms in the roots (Supplementary Table S4), the diversity of all varieties (Shannon: R26, 2.75, R49, 2.93, S44, 2.48, S45, 2.71) was lower in inoculated plants than in uninoculated plants (Shannon: CK26, 3.01; CK49, 3.09; CK44, 5.50, CK45, 4.71). At the same time, the abundance of microorganisms in the infected susceptible plants (Ace: S44, 420.47, S45, 427.09; Chao: S44, 284.20, S45, 348.36) was lower than that in the uninoculated plants (Ace: CK44, 1150.13, CK45, 760.22; Chao: CK44, 1073.63, CK45, 650.01). In Supplementary Table S5, the diversity and richness of microorganisms in the ear of the resistant varieties of inoculated plants (Shannon: R26, 1.46; Ace, R26, 172.04; Chao: R26, 140.33) were determined to be lower than those of uninoculated plants (Shannon: CK26, 2.39; Ace: CK26, 239.29; Chao: CK26, 195.44). In contrast, the microbial diversity and richness of infected plants (Shannon: S45, 4.42; Ace: S45, 1017.29; Chao: S45, 907.69) were observed to be higher than those of uninfected plants (Shannon: CK45, 3.32; Ace: CK45, 487.22; Chao: CK45, 468.45). In the first stem under the ear (Supplementary Table S6), the microbial diversity and richness of the inoculated resistant varieties and susceptible varieties (Shannon: R26, 2.97, S45, 4.84; Ace: R26, 441.79, S45, 1017.34; Chao: R26, 322.75, S45, 986.71) were higher than those of the control plants (Shannon: CK26, 2.80, CK45, 4.12; Ace: CK26, 351.23, CK45, 678.29; Chao: CK26, 279.66, CK45, 591.47). In contrast, for the base of the stem (Supplementary Table S7), the microbial diversity and richness of the inoculated resistant and susceptible plants (Shannon: R26, 3.62, S45, 3.24; Ace: R26, 595.92, S45, 393.89; Chao: R26, 507.42, S45, 319.48) were lower than those of the control plants (Shannon: CK26, 4.13, CK45, 3.40; Ace: CK26, 711.23, CK45, 400.34; Chao: CK26, 651.38, CK45, 350.42). In Supplementary Fig. S3, we performed statistical analysis on the diversity index of the endophytic bacteria of Mianyang 26/Yumai 47 and A-45, and we observed that in the resistant variety Mianyang 26/Yumai 47, there were significant differences between the three diversity index (Shannon, Ace, and Chao) values of the ears and stem bases, and the Shannon index also showed significant difference between the ear and the first stems under the ear. However, there was no significant difference among the three diversity indexes of endophytic bacteria in the susceptible variety A-45. Therefore, the impact of *T. controversa* on wheat endophyte communities differs among different tissue components and plant varieties.

### Microbial community of wheat rhizosphere soil

**Figure 1:**
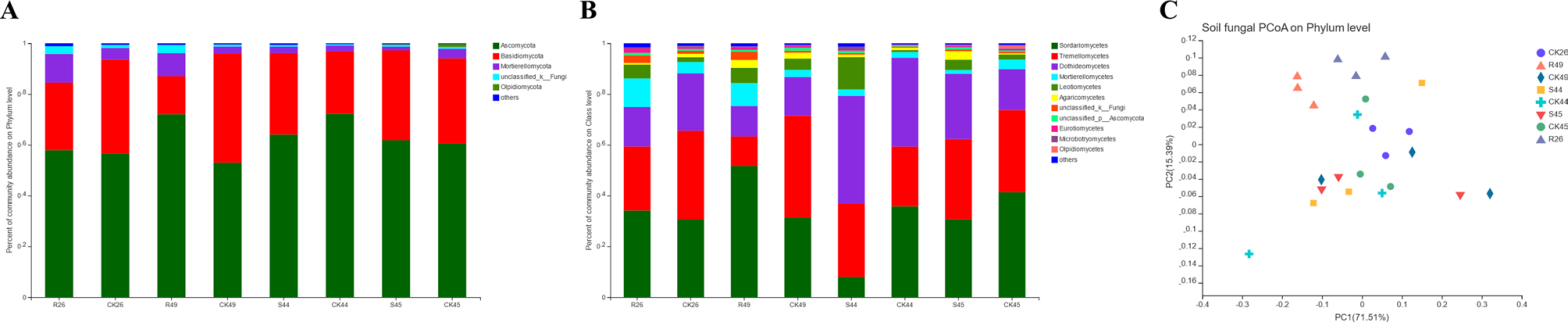
Taxonomic composition of fungal communities in soil at different levels. (**A**) Taxonomic composition of fungal communities at the phylum level, (**B**) Taxonomic composition of fungal communities at the class level, (**C**) Principal coordinate analysis (PCoA) based on Bray–Curtis metrics for fungal communities in soil. ‘Others’ represents the phyla and classes with relative abundances less than 1% of the total sequences. R, resistant variety inoculated with *T. controversa*; S, susceptible variety inoculated with *T. controversa*; CK, wheat not inoculated with *T. controversa*; 26, wheat hybrid variety Mianyang 26/Yumai 47; 49, wheat hybrid variety Yumai 49 * 4/Lankao dwarf 8; 44, wheat variety A-44; 45, wheat variety A-45.

The fungal community in the soil (Fig. 1), as shown in Fig. 1A, mainly consisted of three phyla: Ascomycota, Basidiomycota and Mortierellomycota. The abundance of Mortierellomycota in inoculated Yumai 49 * 4/Lankao dwarf 8 and Mianyang 26/Yumai 47 was higher than that in the susceptible varieties (inoculated and control) and the control resistant variety. In Fig. 1B, the main classes of all samples were Sordariomycetes, Tremellomycetes and Dothideomycetes. We also found that the abundance of Mortierellomycetes was higher in resistant infected varieties Specifically, for Mianyang 26/Yumai 47 and Yumai 49 * 4/Lankao dwarf 8 inoculated with *T. controversa*, the abundances of Sordariomycetes, Mortierellomycetes and Leotiomycetes were higher, while the abundances of Tremellomycetes and Dothideomycetes were lower. For A-44, the abundance of Sordariomycetes in the inoculated plants was lower than that in the uninoculated plants, while the abundances of Tremellomycetes, Dothideomycetes and Leotiomycetes in the inoculated plants were higher than that in the uninoculated plants. Similarly, for A-45, the abundances of Dothideomycetes and Leotiomycetes were higher in the inoculated plants, and the abundances of Sordariomycetes and Mortierellomycetes were higher in the uninoculated plants. After the inoculation of the resistant varieties, the abundance of Sordariomycetes increased compared to the control, while after the inoculation of the susceptible varieties, the abundance of Sordariomycetes decreased compared to the control. Among the resistant varieties, the abundances of Sordariomycetes and Mortierellomycetes were higher after *T. controversa* infection than control plants. However, in the susceptible varieties, compared with the control, the abundance of Dothideomycetes increased after *T. controversa* infection. The abundance of Leotiomycetes in inoculated susceptible and resistant varieties was increased, indicating that Leotiomycetes was a useful indicator of pathogen infection. In Fig. 1C, the fungal community diversity is shown. Bray–Curtis metrics and principal coordinate analysis (PCoA) showed that resistant varieties of Mianyang 26/Yumai 47 and Yumai 49 * 4/Lankao dwarf 8 form two clusters after inoculation with *T. controversa*, showing that the diversity of the microbial community changes after inoculation with *T. controversa*.

**Figure 2:**
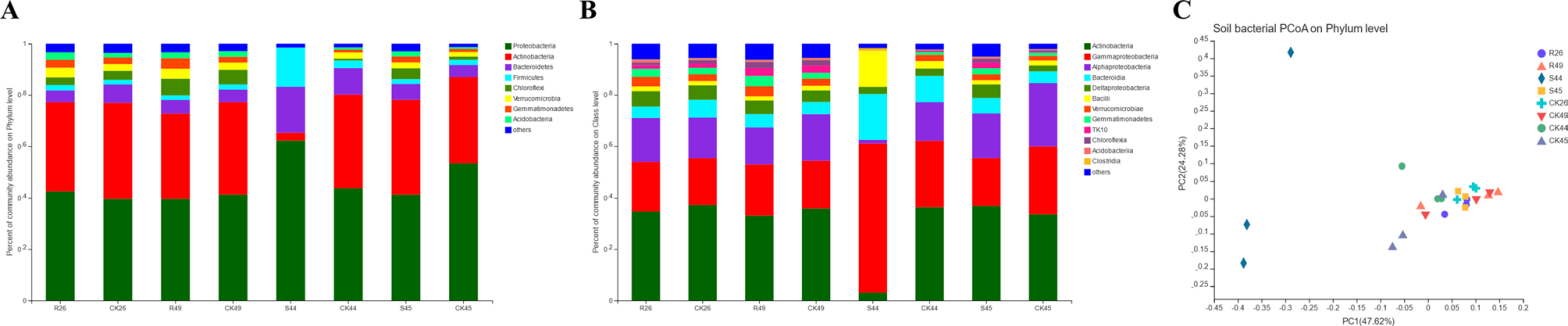
Taxonomic composition of bacterial communities in soil at different levels. (**A**) Taxonomic composition of bacterial communities at the phylum level, (**B**) Taxonomic composition of bacterial communities at the class level, (**C**) Principal coordinate analysis (PCoA) based on Bray–Curtis metrics for bacterial communities in soil. ‘Others’ represents the phyla and classes with relative abundances less than 1% of the total sequences. R, resistant variety inoculated with *T. controversa*; S, susceptible variety inoculated with *T. controversa*; CK, wheat not inoculated with *T. controversa*; 26, wheat hybrid variety Mianyang 26/Yumai 47; 49, wheat hybrid variety Yumai 49 * 4/Lankao dwarf 8; 44, wheat variety A-44; 45, wheat variety A-45.

The bacterial community in the soil (Fig. 2) was more diverse than the fungal community. As shown in Fig. 2A, Proteobacteria, Actinobacteria, Bacteroidetes, Firmicutes, Chloroflexi, Verrucomicrobia, Gammaproteobacteria and Acidobacteria were the main components of the microbial community. The abundance of Proteobacteria, Firmicutes and Bacteroidetes in inoculated A-44 was higher than that in plants with the other seven treatments, while Actinobacteria exhibited the lowest abundance, and no Chloroflexi was found. As shown in Fig. 2B, the dominant classes in all soil community samples were Actinobacteria, Gammaproteobacteria, Alphaproteobacteria, Bacteroidia, Deltaproteobacteria, Bacilli, Verrucomicrobiae, Gemmatimonadetes, TK10 and Chloroflexia. Compared with the control, the abundance of bacteria in the communities of inoculated resistant varieties Mianyang 26/Yumai 47 and Yumai 49 * 4/Lankao dwarf 8 did not exhibit notable changes. For A-44, the bacterial community in the soil of the inoculated plants changed compared to the control. The abundance of Actinobacteria and Alphaproteobacteria in the inoculated plants was lower than that in the uninoculated plants, and Gammaproteobacteria, Bacteroidia and Bacilli were much more abundant in the inoculated plants than in the uninoculated plants. In A-45, the abundance of Actinobacteria, Bacteroidia, Deltaproteobacteria, Gemmatimonadetes and TK10 was higher in inoculated plants, and the abundance of Gammaproteobacteria and Alphaproteobacteria was lower in inoculated plants, compared with uninoculated plants. The abundance of Actinobacteria decreased in infected resistant cultivars, while the abundance of Bacteroidia increased in infected susceptible cultivars. In Fig. 2C, a study of the differences between PCoA groups showed that uninoculated A-44 and A-45 formed a cluster and separated from other groups. At the same time, there were clear differences in the diversity of inoculated A-44. This finding shows that the diversity of the bacterial community in the rhizosphere soil of the normally susceptible wheat variety is different from that of the inoculated susceptible wheat variety, and it is also different from that of the normal resistant variety.

### Composition and difference in entophytic bacteria in tissues of wheat

**Figure 3:**
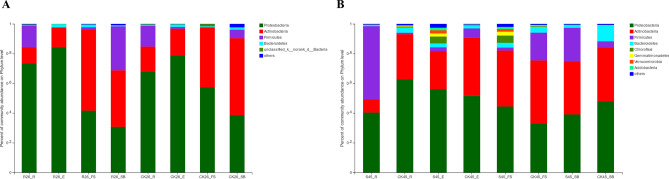
Taxonomic composition of the endogenous bacteria in the roots (R), stem base (SB), first stem under the ear (FS) and ear (E) of two wheat varieties. (**A**) The taxonomic composition of the disease-resistant variety Mianyang 26/Yumai 47 at the phylum level; (**B**) The taxonomic composition of the susceptible variety A-45 at the phylum level. ‘Others’ represents the phyla and classes with relative abundances less than 1% of the total sequences. R, resistant variety inoculated with *T. controversa*; S, susceptible variety inoculated with *T. controversa*; CK, wheat not inoculated with *T. controversa*; 26, wheat hybrid variety Mianyang 26/Yumai 47; 45, wheat variety A-45.

We observed that Proteobacteria, Firmicutes and Actinobacteria were the dominant bacteria in wheat tissue samples (Fig. 3). The abundance of Proteobacteria in the roots and ears of Mianyang 26/Yumai 47 was higher than that in controls, while it was decreased in the first stem under the ear and stem base compared with the controls (Fig. 3A). The stem base had a higher abundance of Firmicutes than the control. Except for the first stem under the ear, the abundance of Actinobacteria in other tissues was decreased in the inoculated plants (Fig. 3A). The diversity of wheat roots, ears and the first stem under the ears was higher in the susceptible variety A-45 than in the resistant variety (Fig. 3B). The roots of the inoculated wheat exhibited the lowest abundance of Actinobacteria and the highest abundance of Firmicutes in all treatments. The abundances of Chloroflexi, Gemmatimonadetes, Verrucomicrobia and Acidobacteria in the ear and the first stem under the ear of A-45 were considerably higher than those in other organs (Fig. 3B).

**Figure 4:**
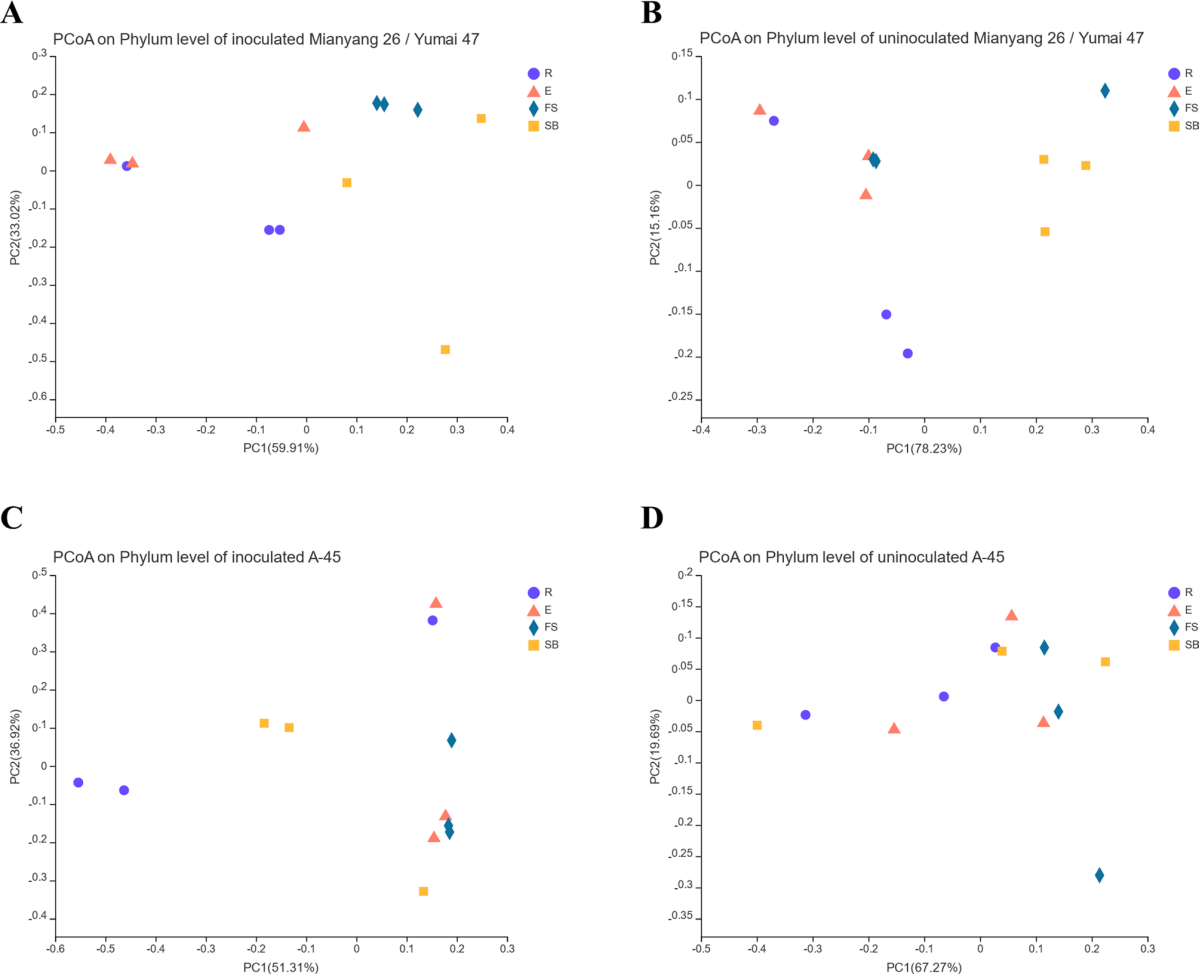
Bray–Curtis metrics-based principal coordinate analysis (PCoA) analysis of the endophytic bacteria (phylum level) in various tissues of different wheat varieties. (**A**) Analysis of differences between groups of endophytes in Mianyang 26/Yumai 47 inoculated with *T. controversa*. (**B**) Analysis of differences between groups of endophytes in Mianyang 26/Yumai 47 not inoculated with *T. controversa*. (**C**) Analysis of the difference between the endophytes in A-45 inoculated with *T. controversa.* (**D**) Analysis of differences between groups of endophytes in A-45 not inoculated with *T. controversa*. R, root; E, ear; FS, first stem under the ear; SB, stem base.

We applied principal coordinate analysis (PCoA) and Bray–Curtis metrics to compare the differences and similarities in the bacterial community diversity between the resistant variety Mianyang 26/Yumai 47 and the susceptible variety A-45 (Fig. 4). For the inoculated Mianyang 26/Yumai 47 plants, the first and second principal component analysis variables (PC1 was 59.91% and PC2 was 33.02%) accounted for 92.93% of the cumulative variance of all samples (Fig. 4A). There were significant differences in the microbial composition of the roots, spikes, first stem under the ear and base of the stem. Compared with the other treatments, the FS group had significant differences. For the uninoculated Mianyang 26/Yumai 47 plants, PC1 was 78.23% and PC2 was 15.16%, accounting for 93.39% of the cumulative variance of all samples (Fig. 4B). There were large differences between the groups, and the SB group yielded better results. There were also significant differences between groups in inoculated A-45 (Fig. 4C) and uninoculated A-45 (Fig. 4D). The results showed that regardless of whether resistant varieties or susceptible varieties were present, the microbial compositions of wheat roots, ears, first stem under the ear and base of the stems were different.

**Figure 5:**
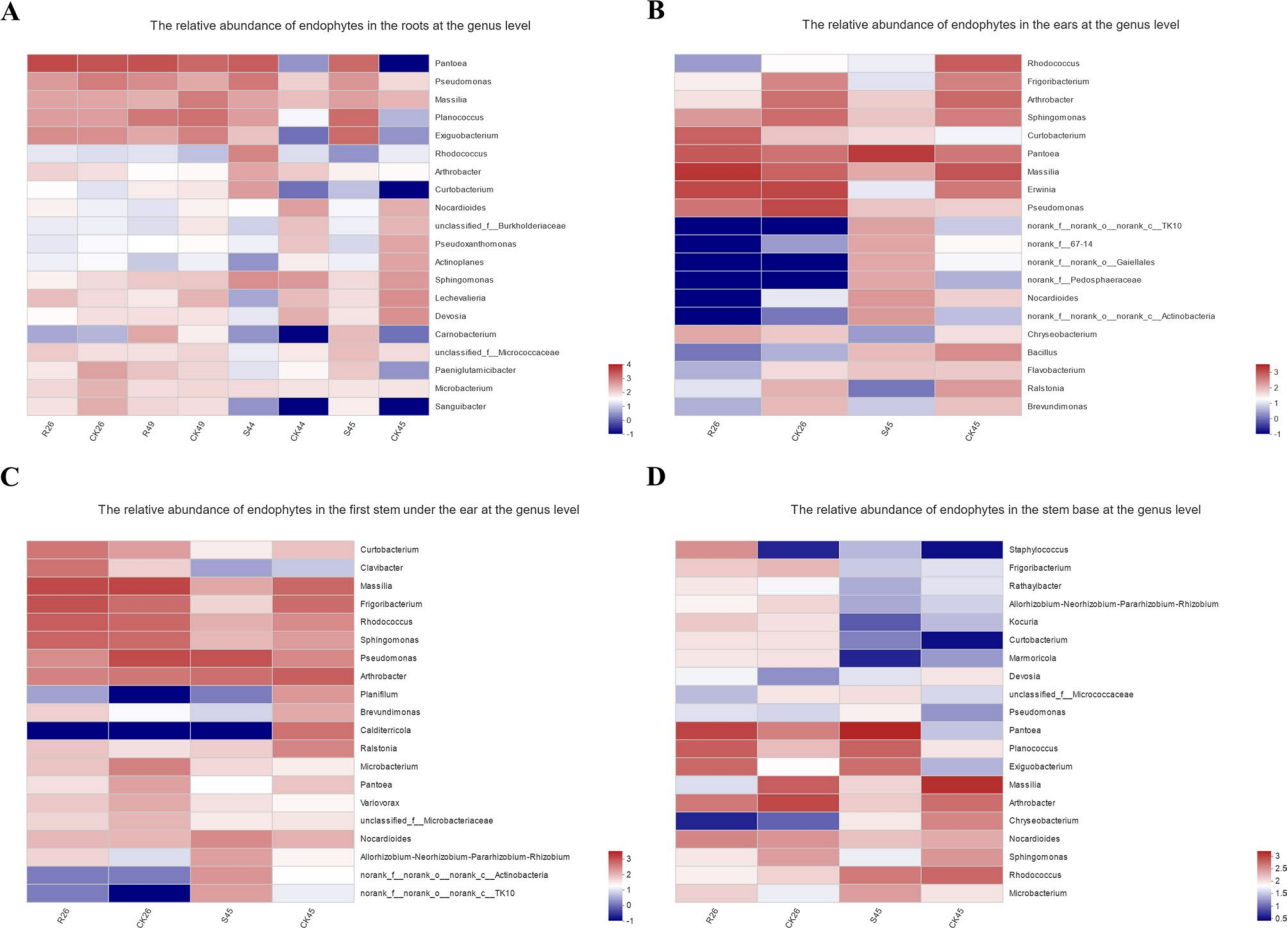
Heatmap of the relative abundance of endophytic bacteria in different tissues of wheat. (**A**) The relative abundance of endophytes in the roots at the genus level. (**B**) The relative abundance of endophytes in the ears at the genus level. (**C**) The relative abundance of endophytes in the first stem under the ear at the genus level. (**D**) The relative abundance of endophytes in the stem base at the genus level. R, resistant variety inoculated with *T. controversa*; S, susceptible variety inoculated with *T. controversa*; CK, wheat not inoculated with *T. controversa*; 26, wheat hybrid variety Mianyang 26/Yumai 47; 49, wheat hybrid variety Yumai 49 * 4/Lankao dwarf 8; 44, wheat variety A-44; 45, wheat variety A-45. The analysis were performed by vegan: Community Ecology Package. R package version 2.5–5^43^. https://CRAN.R-project.org/package=vegan.

We also used a heatmap to explore the species composition of microbes in wheat at the genus level and selected the top 20 most abundant species (Fig. 5). As shown in Fig. 5A, at the roots of wheat, there were high abundances of *Pseudomonas*, *Massilia* and *Microbacterium* in all samples. The abundances of *Exiguobacterium*, *Planococcus* and *Pantoea* in the uninfected susceptible varieties were lower than those in the other treatment groups. Compared with uninoculated wheat, we observed that the abundances of *Lechevalieria*, *Nocardioides* and *Arthrobacter* in Mianyang 26/Yumai 47 were increased, and the abundances of *Paeniglutamicibacter*, *Carnobacterium*, *Planococcus*, *Pseudomonas* and *Pantoea* in Yumai 49 * 4/Lankao dwarf 8 were increased. In inoculated A-44, the abundances of *Sanguibacter*, *Microbacterium* and *Carnobacterium* and other bacterial groups with low abundance increased slightly compared with the control. The abundances of *Sphingomonas*, *Curtobacterium*, *Arthrobacter*, *Rhodococcus*, *Exiguobacterium*, *Planococcus*, *Massilia*, *Pseudomonas* and *Pantoea* were all increased. For A-45, the change trend of the microflora was mostly consistent with A-44, except that the abundance of *Rhodococcus* and *Sphingomonas* was reduced, and that of *Paeniglutamicibacter* was increased, in the inoculated plants. As shown in Fig. 5B, for the microbial flora of wheat ears, we found that the abundance of *Chryseobacterium*, *Massilia*, *Pantoea* and *Curtobacterium* in the resistant varieties Mianyang 26/Yumai 47 was higher in the inoculated plants. In the susceptible variety A-45, the abundance of *Nocardioides*, *Pseudomonas*, *Pantoea* and *Curtobacterium* was higher in the inoculated wheat ears than in the uninoculated wheat ears. As shown in Fig. 5C, in the first stem under the ear, compared with uninoculated wheat, *Sphingomonas*, *Rhodococcus*, *Frigoribacterium*, *Allorhizobium-Neorhizobium-Pararhizobium-Rrhizobium*, *Clavibacter* and *Curtobacterium* were more abundant in inoculated Mianyang 26/Yumai 47 plants, while in inoculated A-45 plants, the abundances of *Allorhizobium-Neorhizobium-Pararhizobium-Rrhizobium*, *Nocardioides*, *Variovorax*, *Microbacterium* and *Pseudomonas* were higher. As shown in Fig. 5D, at the base of the stem, the abundances of *Exiguobacterium*, *Planococcus* and *Pantoea* in uninfected susceptible varieties were lower than in the other treatments. The abundances of *Microbacterium*, *Nocardioides*, *Exiguobacterium*, *Planococcus*, *Pantoea*, *Kocuria*, *Rathayibacter* and *Staphylococcus* in the inoculated Mianyang 26/Yumai 47 plants were higher than in the uninoculated plants. The trend of A-45 was largely consistent with the resistant varieties, but the changes in some low-abundance microbial communities, such as *Kocuria* and *Rathayibacter*, were opposite to the changes observed in the microbial communities of Mianyang 26/Yumai 47.

### Network analysis

**Figure 6:**
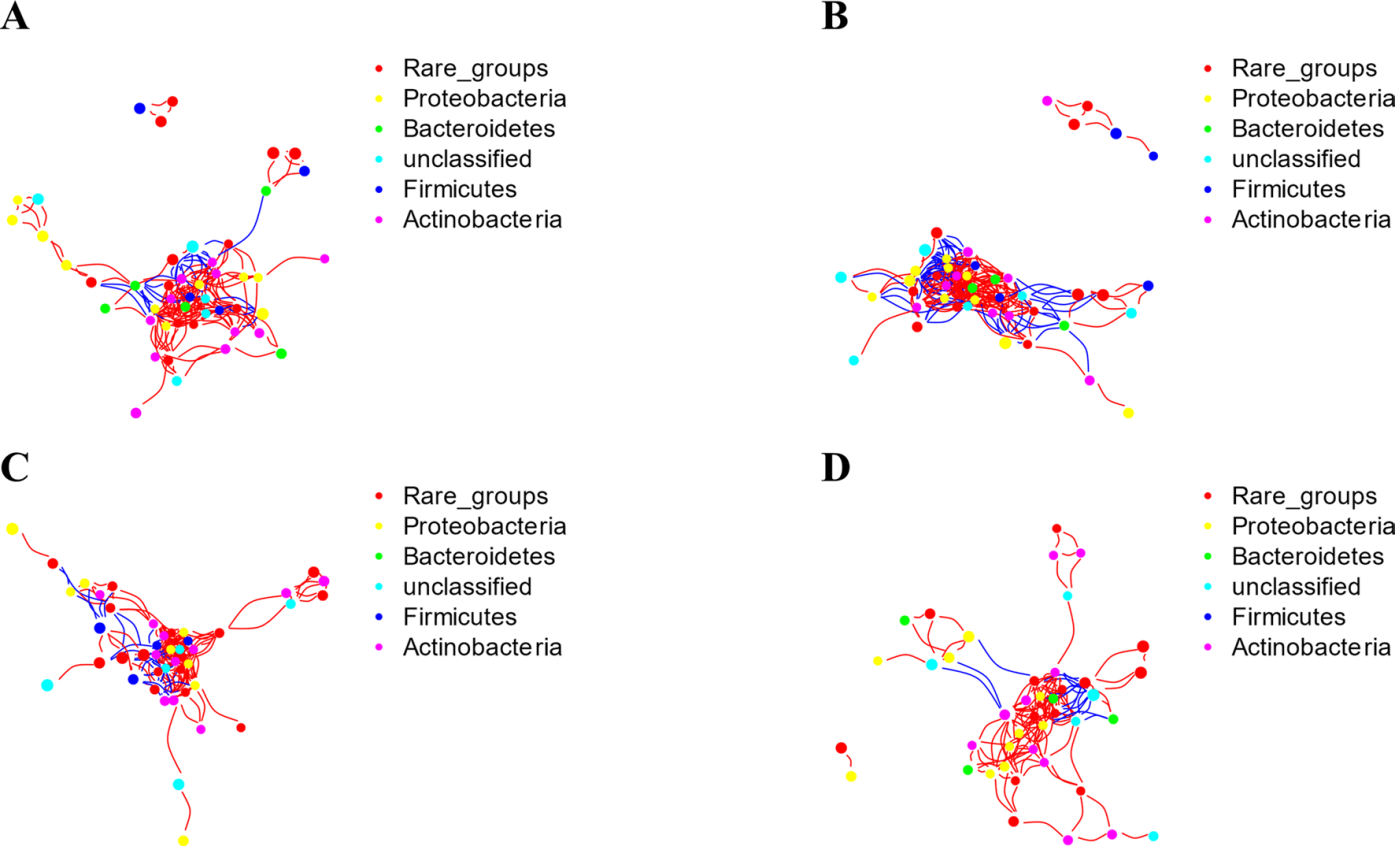
Bacterial coexpression network analysis of healthy wheat and inoculated wheat of resistant and susceptible wheat varieties. (**A**) Coexpression network of inoculated resistant wheat. (**B**) Coexpression network of healthy resistant wheat. (**C**) Coexpression network of inoculated susceptible wheat. (**D**) Healthy susceptible wheat coexpression network. Positive interactions are depicted as red edges, and the negative interactions are depicted as blue edges. Descriptive and topological network properties were performed with the R package igraph^44^. The co-occurrence networks were using the “Fruchterman-Reingold” layout with 10^4^ permutations in igraph^45^.

**Figure 7:**
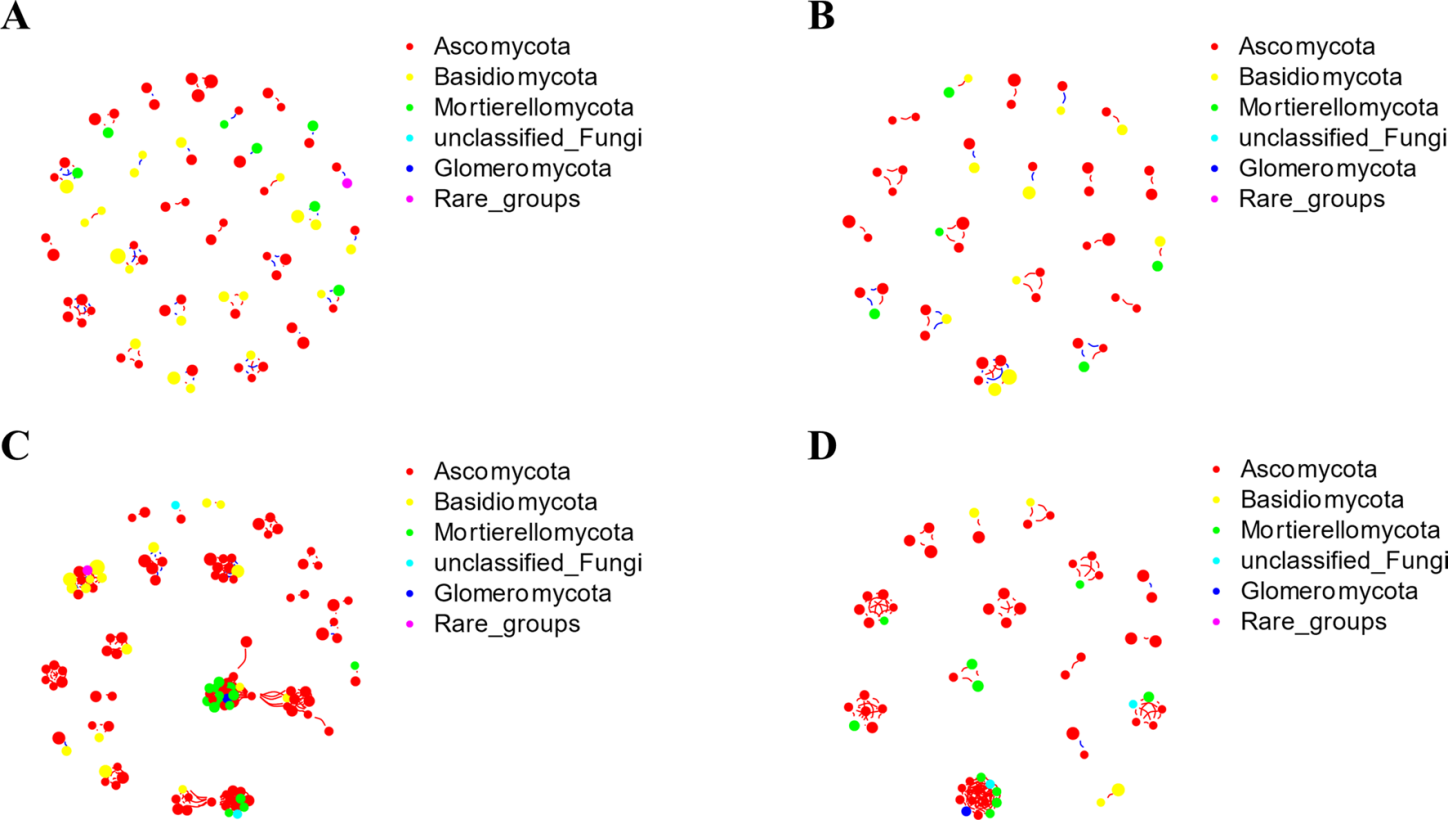
Fungal coexpression network analysis of healthy wheat and inoculated wheat of resistant and susceptible wheat varieties. (**A**) Coexpression network of inoculated resistant wheat. (**B**) Coexpression network of healthy resistant wheat. (**C**) Coexpression network of inoculated susceptible wheat. (**D**) Coexpression network of healthy susceptible wheat. Positive interactions are depicted as red edges, and the negative interactions are depicted as blue edges. Descriptive and topological network properties were performed with the R package igraph^44^. The co-occurrence networks were using the “Fruchterman-Reingold” layout with 10^4^ permutations in igraph^45^.

We established a coexpression network relationship between healthy wheat and diseased wheat to visualize the relationship between samples and microorganisms. According to taxonomic annotations, the bacterial microorganisms in all groups were mainly composed of Proteobacteria, Bacteroidetes, Firmicutes and Actinobacteria (Fig. 6). Among resistant wheat, healthy wheat had higher connectivity (CK: 0.29; R: 0.19) and average degree (CK: 12.89; R: 8.51). In contrast, among susceptible wheat, the connectance (CK: 0.18; R: 0.24) and average degree (CK: 7.48; R: 10.32) of diseased wheat were higher (Supplementary Table S8). We also performed taxonomic annotations on fungi. The fungal microorganisms in all groups were mainly composed of Ascomycota, Basidiomycota, Glomeromycota and Mortierellomycota (Fig. 7). We found that there were more edges in the susceptible varieties than in the resistant varieties, indicating that the fungal interaction network in the susceptible varieties was more abundant (Supplementary Table S9).

**Figure 8:**
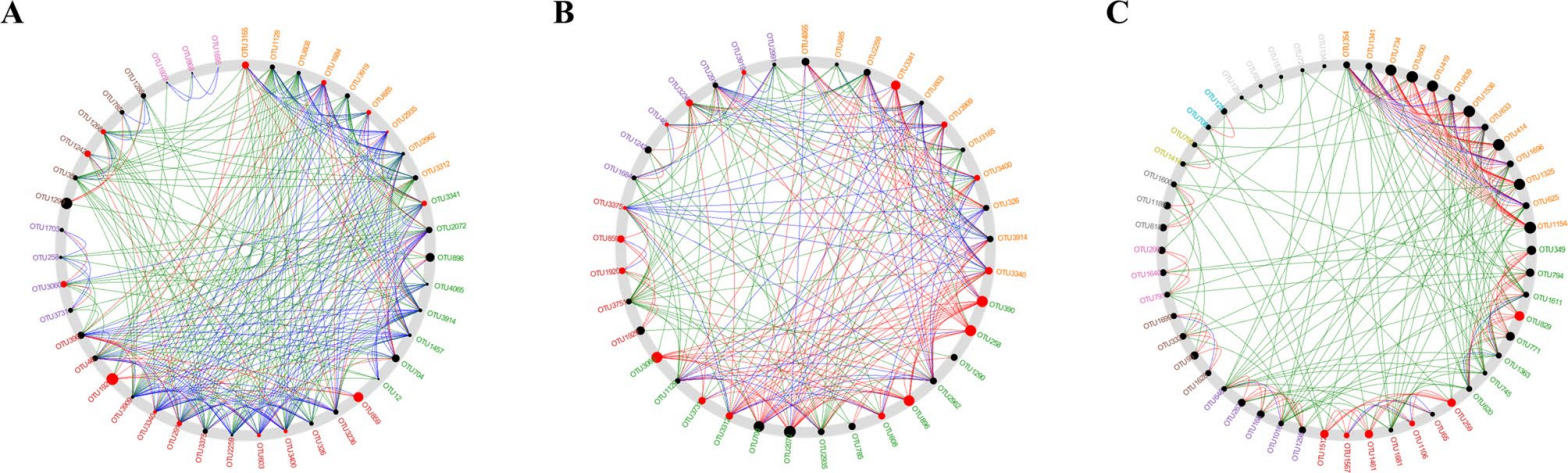
Analysis of potential taxa based on the network analysis of fungi and bacteria in inoculated and uninoculated wheat. (**A**) Potential taxa based on bacterial network analysis of inoculated and uninoculated resistant wheat. (**B**) Potential taxa based on bacterial network analysis of inoculated and uninoculated susceptible wheat. (**C**) Potential taxa based on fungal network analysis of inoculated and uninoculated resistant wheat. Red and large nodes are important "drivers", and grey out nodes represent nodes that exist simultaneously in the inoculated and uninoculated resistant wheat microbial community but directly interact with the common subnetwork. Red edges indicate associations present only in uninoculated wheat microbiomes, green edges indicate associations present only in inoculated wheat microbiomes, and blue edges indicate associations present in both inoculated and uninoculated wheat microbiomes. The NetShift method (https://web.rniapps.net/netshift) was used to identify the key taxa between healthy wheat and inoculated wheat microbial communities^27, 28^.

The NetShift method (https://web.rniapps.net/netshift) was further utilized to identify the key taxa in the network according to the network interaction differences between healthy wheat and inoculated wheat microbial communities^27, 28^. Figure 8A shows that in resistant wheat, the potential bacterial flora related to the interaction of wheat and *T. controversa* primarily consisted of *Massilia*, *Acidovorax*, *Pseudoxanthomonas*, *MND1*, *Paeniglutamicibacter*, *Altererythrobacter*, *Sphingomona, Acidibacter*, Micrococcaceae, *Erwinia*, *Curtobacterium*, and *Frigoribacterium*, and our data did not capture the potential fungal flora. Figure 8B,C showed that in susceptible wheat, the important bacterial "drivers" in the interaction between wheat and *T. controversa* were *MND1*, Xanthomonadaceae, *Altererythrobacter*, *Sphingomonas*, Actinobacteria, *Pantoea*, *Nocardioides*, *Devosia*, *Phyllobacterium*, *Erwinia*, Micrococcaceae, *Paeniglutamicibacter*, *Lechevalieria*, *Flavobacterium*, and *Massilia*. The potential fungal "drivers" primarily consisted of Hypocreales, *Monodictys*, *Ilyonectria*, *Fusarium*, and Nectriaceae.

## Discussion

In this study, the microbial communities of wheat varieties resistant and susceptible to dwarf bunt, including wheat tissues of roots, ears, first stem under the ear, and stem base, as well as the microbial community in rhizosphere soil, were analysed. The results may facilitate the control of dwarf bunt in wheat and may establish a foundation for future research exploring the interactions among plant, pathogen and microbial communities.

### Composition and diversity of microorganisms in rhizosphere soil

Through the study of soil fungal communities, we observed that the abundance of Ascomycota and Basidiomycota detected in this study was higher than that of other fungi, and this phenomenon was also observed in strawberry^29^. Second, we determined that in both inoculated plants and control plants, the richness of fungal communities in resistant varieties was higher than that in susceptible varieties. A richer and more diverse community leads to increased competition for resources between species, which has been described as a key factor determining whether pathogens can successfully invade plants^30^.

For the bacterial community in soil samples, Proteobacteria and Actinobacteria were most abundant, and this result was also reported in rice^31^, soybean^32^, and grape^33^. Many members of Proteobacteria can produce important substances that enable bacteria to be adsorbed onto the surfaces of plants and plant roots and promote interactions^34^.

### Composition and diversity of endophytic bacteria in wheat tissues

We detected bacteria in wheat tissues of roots, ears, first stem under the ear, and stem base. In our research, Proteobacteria, Firmicutes and Actinobacteria were the predominant bacteria in wheat, which was also reported by Robinson et al.^35^ and Wemheuer et al.^36^. Proteobacteria and Firmicutes were the two most abundant phyla in healthy wheat and grasses, respectively. We also observed that there was no significant difference between the diversity and richness of susceptible varieties and resistant varieties. However, Xu et al.^37^ reported that the diversity of the bacterial communities of resistant varieties was higher than that of susceptible varieties in mulberry trees. Based on the heatmap, the abundance of *Exiguobacterium*, *Planococcus* and *Pantoea* associated with susceptible varieties was lower than that in the other treatment groups in the root and stem base.

The results of this study showed that the microbial diversity of bacteria in the rhizosphere was higher than that in the roots which, as demonstrated by the root surface of plants, has a gating effect and is selective for the entry of bacteria^38^. In general, endophytic microbial communities are more specific than rhizosphere soil microbial communities because plants allow less adaptive bacteria to enter and survive in plants^39^. These endophytic microorganisms can interact with plants and have antagonistic effects on pathogens^40^.

### Potential active microbial community in wheat tissues and rhizosphere soil

In this study, the abundances of some microbial populations in the susceptible and resistant varieties increased after *T. controversa* inoculation. For example, in the rhizosphere soil, after inoculation with *T. controversa*, the abundances of Sordariomycetes and Mortierellomycetes in resistant varieties increased compared with those in the control. In infected susceptible plants, the abundances of Dothideomycetes and Bacteroidia increased after inoculation. In the resistant and susceptible varieties, after inoculation, the abundance of Leotiomycetes increased.

The root is an important organ for the entry of soil microorganisms into the plant, and the ear is targeted by *T. controversa* after infection. Therefore, we focused on the changes in microorganisms in these two plant tissues to further characterize the role played by microbial communities in dwarf bunt-resistant wheat varieties. In wheat tissues, we found large differences in the microbial composition of different parts of wheat, indicating that the potential indicator flora was also different. In the wheat roots, we found that the abundances of bacteria in the two groups of resistant varieties were different after inoculation. However, the changes in bacterial populations in the two susceptible varieties were more consistent. In susceptible wheat infected by *T. controversa*, the microbial communities in the ear and the first section under the ear undergo similar changes. The abundances of Chloroflexi, Bacteroidetes, Gemmatimonadetes, Verrucomicrobia and Acidobacteria in the ear and the first section under the ear of susceptible plants were higher than those in other tissues and were higher than those in resistant plants. This result indicated that the bacteria of these phyla could be associated with the formation of wheat disease spikes. Further analysis of the changes in bacteria in wheat ears at the genus level demonstrated that in both resistant and susceptible varieties, the abundance of *Pantoea* and *Curtobacterium* was higher in the inoculated plants; therefore, these two genera could be useful indicators for *T. controversa*. Notably, the abundances of *Chryseobacterium* and *Massilia* in the spikes of the resistant varieties were higher than that of the uninoculated wheat, while the abundances of *Nocardioides* and *Pseudomonas* were higher in the susceptible varieties.

The co-occurrence network analysis of the microbial communities of plants and soil may be employed to strengthen disease management and identify candidate microorganisms that affect plant health. Based on NetShift analysis, we found some potential microorganisms related to wheat-*T. controversa* interactions. Most of these microorganisms or their classes have been discussed above to further characterize their effects, such as *Massilia, Sphingomonas, Paeniglutamicibacter, Curtobacterium*, *Frigoribacterium, Pantoea*, *Nocardioides*, *Lechevalieria,* Alphaproteobacteria, Actinobacteria, Bacteroidia, Sordariomycetes and Dothideomycetes.

In summary, Sordariomycetes, Mortierellomycetes, Leotiomycetes, *Chryseobacterium* and *Massilia* were determined to be more abundant in inoculated resistant varieties than in control plants (Figs. 2, 6), and Dothideomycetes, Bacteroidia, *Nocardioides* and *Pseudomonas* were observed to be more abundant in susceptible varieties after infection with *T. controversa* compared with the control (Figs. 2, 3, 6), and *Curtobacterium*, *Exiguobacterium*, *Planococcus*, and *Pantoea* exhibited higher abundances in both the susceptible and resistant varieties after inoculation than in the controls (Fig. 6).

## Materials and methods

### Source and inoculation of fungal materials

*T. controversa* was provided by Blair Goates, United States Department of Agriculture (USDA), Agricultural Research Service (ARS), Aberdeen, Idaho, USA. The soil mix (Klasmann-deilmann, Germany) was placed in an autoclave (ZEALWAY, GR110DA, China) and autoclaved at 121 °C for 30 min to kill microorganisms and insects. Seeds were seeded in a 20-cm-diameter pot with sterilized soil at a depth of approximately 3 cm, with 15 seeds in each pot. The seeds were cultured in an ultralow temperature biochemical incubator (Percival, ARC-36VL-lt, USA) for 24 h under full light with a light intensity of 300 mol/m^2^/s. After sowing, the soil surface was covered with teliospores at a density of 3 × 10^6^/m^2^. At the initial stage of seedlings, the seeds were allowed to germinate and grow to the one-leaf stage (Z11)^41^ at 4 °C. When the seedlings grew to the jointing stage (Z31), the temperature in the biochemical incubator was adjusted to 18 °C, and when the wheat grew to the booting stage (Z41), the temperature in the biochemical incubator was adjusted to 25 °C.

### Detection of *T. controversa* in wheat leaves

We used a plant genomic DNA extraction kit (TianGen, Beijing, China) to extract DNA from wheat leaves (Z13). The specific primer pairs for detecting *T. controversa* (ISSR859-140AF-5′-TGGTGGTCGGGAAAGATTAGA-3′, ISSR859-511AR: 5′-GGGACGAAGGCATCAAGAAG-3′). PCR amplification procedures were performed based on Gao’s method^42^^.^

### Plant materials and sample collection

Samples of wheat plants (Z92)^41^ and rhizosphere soil from 4 varieties (three plants per variety) were collected from a greenhouse (Table 1). We pooled the three replicates for sequencing. The resistant winter wheat varieties were Mianyang 26/Yumai 47 and Yumai 49 * 4/Lankao dwarf 8, and the susceptible winter wheat varieties were A-44 and A-45. Wheat seeds were obtained from the Institute of Plant Protection, Chinese Academy of Agricultural Sciences Beijing-China. We set up three biological replicates for sequencing.

**Table 1:** Sample collection information.

Varieties	Number of samples				
	Soil	Root	Stem base	First stem of the ear	Ear
Mianyang 26/Yumai 47	12	6	6	6	6
Yumai 49 * 4/Lankao dwarf 8	12	6	0	0	0
A-44	12	6	0	0	0
A-45	12	6	6	6	6

The samples for each variety of wheat were collected from both inoculated and uninoculated plants.

Soil sample collection: wheat plants were carefully collected; large portions of soil were removed from the roots, placed on ice and transported to the laboratory. After shaking the roots to remove loose soil, a sterile brush was employed to collect the residual soil from the roots. Equal amounts of rhizosphere soil from the three wheat plants were mixed and stored at − 80 °C.

Wheat tissue collection: A sterile brush was used to remove residual soil and impurities; sterile water was used to shake and wash the plant fragments multiple times, and the washing solution was centrifuged at 12,000×g for 10 min. The washed plant tissue was cut into small pieces of 0.5–1 cm with sterile scissors, and the pieces were placed in a sterile Eppendorf tube. The wheat plant parts collected included the root, ear, first stem under the ear and base of the stem. All samples were kept at -80 °C for further use.

### DNA extraction and PCR amplification

Total soil and plant tissue DNA were extracted using a FastDNA SPIN Soil kit (MP Biomedicals, Solon, USA). A NanoDrop 2000 UV–vis spectrophotometer (Thermo Scientific, Wilmington, USA) was utilized to determine the DNA concentration and purity, and 1% agarose gel electrophoresis was used to determine the DNA extraction quality. The primers used to amplify the hypervariable regions V5-V7 of the bacterial 16S rRNA gene were 799F (5′-AACMGGATTAGATACCCKG-3′) and 1193R (5′-ACGTCATCCCCACCTTCC-3′). The primers used to amplify the internal transcribed spacer (ITS) region of ribosomal DNA were ITS1F (5′-CTTGGTCATTTAGAGGAAGTAA-3′) and ITS2R (5′-GCTGCGTTCTTCATCGATGC-3′). The programme for PCR amplification was as follows: initial denaturation at 95 °C for 3 min; 27 cycles of denaturing at 95 °C for 30 s, annealing at 55 °C for 30 s and extension at 72 °C for 45 s; a single extension at 72 °C for 10 min; and a final extension at 4 °C. The reaction system included 4 μL 5 × FastPfu buffer, 2 μL 2.5 mM dNTPs, 0.8 μL forward primer (5 μM), 0.8 μL reverse primer (5 μM), 0.4 μL FastPfu Polymerase, 0.2 μL BSA, 10 ng template DNA, and ddH_2_O up to 20 μL. The reactions were performed in triplicate. The PCR product was extracted from a 2% agarose gel and purified with an AxyPrep DNA Gel Extraction Kit (Axygen Biosciences, Union City, CA, USA) based on the manufacturer's instructions and quantified using a Quantus Fluorometer (Promega, USA).

### Illumina MiSeq sequencing and data analysis

Based on the standard protocols by Majorbio Bio-Pharm Technology Co. Ltd. (Shanghai, China), we used an Illumina MiSeq platform (Illumina, San Diego, USA) to pool purified amplicons in equimolar amounts and performed paired-end sequencing (2 × 300).

The original sequencing data were processed by Trimmomatic software and spliced by FLASH software: (i) the 300-bp reads were truncated at any site with an average quality score of < 20 over a 50-bp sliding window, and the truncated reads shorter than 50 bp were discarded. Reads containing ambiguous characters were also discarded; (ii) only overlapping sequences longer than 10 bp were assembled according to their overlapping sequence. The maximum mismatch ratio of the overlap region was 0.2. Reads that could not be assembled were discarded. (iii) Samples were distinguished according to the barcode and primers, and the sequence direction was adjusted for exact barcode matching and 2 nucleotide mismatches in primer matching.

Using UPARSE software (version 7.1 http://drive5.com/uparse/), OTUs were clustered based on 97% similarity, and single sequences and chimaeras were removed during the clustering process. The RDP classifier (http://rdp.cme.msu.edu/) was utilized to classify and annotate each sequence. The Silva database (SSU123) was utilized to compare 16S rRNA sequences, and Unite (Release 6.0 http://unite.ut.ee/index.php) was utilized to compare the internal transcribed spacer region; the confidence threshold was 0.7. All raw paired-end Illumina sequence data have been deposited in the National Center for Biotechnology Information (NCBI) Sequence Read Archive database (https://www.ncbi.nlm.nih.gov/sra) under BioProject no. PRJNA639912.

## Supplementary Information

Supplementary Information 1.

Supplementary Figure 1.

Supplementary Figure 2.

Supplementary Figure 2.

Supplementary Table 1.

Supplementary Table 2.

Supplementary Table 3.

Supplementary Table 4.

Supplementary Table 5.

Supplementary Table 6.

Supplementary Table 7.

Supplementary Table 8.

Supplementary Table 9.
