# Supplementary material for: Characterization of the microbial communities in wheat tissues and rhizosphere soil caused by dwarf bunt of wheat
Source: Sci Rep. 2021 Mar 11;11:5773. doi: 10.1038/s41598-021-85281-8 (PMC7952392; doi:10.1038/s41598-021-85281-8)
Supplement: Supplementary file 1 — Supplementary Information 1. [file 41598_2021_85281_MOESM1_ESM.docx]

Title page for Supplementary Information

**Characterization of the** **microbial communities in wheat tissues and rhizosphere soil caused by dwarf bunt of wheat**

**Tongshuo Xu^1^#, Wenli Jiang^1,2^**#, D**andan Qin^1^#, Taiguo Liu^1^,** **Jianmin Zhang^2^, Wanquan Chen^1^, Li Gao^1*^**

^1^State Key Laboratory for Biology of Plant Disease and Insect Pests, Institute of Plant Protection, Chinese Academy of Agricultural Sciences, Beijing, China

^2^School of Agriculture, Yangtze University, Jingzhou, China.

Tongshuo Xu^#^, Wenli Jiang^#^, Dandan Qin^#^ contributed equally.

*** Correspondence:** Li Gao ([xiaogaosx@hotmail.com](mailto:xiaogaosx@hotmail.com))

**Supplementary Figure 1.** Molecular detection of *T. controversa* in wheats. M: 2000 DNA marker; 1, 15: DNA from a *T. controversa* teliospore as the positive control; 2-4: DNA from the leaves of inoculated Mianyang 26 / Yumai 47 (Z13); 5-7: DNA from the leaves of uninoculated Mianyang 26 / Yumai 47 (Z13); 8-10: DNA from the leaves of inoculated Yumai 49 * 4 / Lankao dwarf 8 (Z13); 11-13: DNA from the leaves of uninoculated Yumai 49 * 4 / Lankao dwarf 8 (Z13); 16-18: DNA from the leaves of inoculated A-44 (Z13); 19-21: DNA from the leaves of uninoculated A-44 (Z13); 22-24: DNA from the leaves of inoculated A-45 (Z13); 25-27: DNA from the leaves of uninoculated A-45 (Z13); 14,28: sterilized ddH_2_O as the negative control.

**Supplementary Figure 2.** Dilution curves of all wheat samples at the OTU level; the similarity was 97%. (A) Dilution curve of 16S rRNA in soil; (B) Dilution curve of 16S rRNA in roots; (C) Dilution curve of 16S rRNA in the base of the stem; (D) Dilution curve of 16S rRNA in the first stem under the ear; (E) Dilution curve of 16S rRNA in the ear; (F) Dilution curve of the ITS region in soil. R, resistant variety inoculated with *T. controversa*; S, susceptible variety inoculated with *T. controversa*; CK, wheat not inoculated with *T. controversa*; 26, wheat hybrid variety Mianyang 26 / Yumai 47; 49, wheat hybrid variety Yumai 49 * 4 / Lankao dwarf 8; 44, wheat variety A-44; 45, wheat variety A-45.

**Supplementary Figure 3.** Characterization of the microbial richness and diversity index of wheat tissues. (A) Shannon index of OTU level of inoculated Mianyang 26 / Yumai 47; (B) Ace index of OTU level of inoculated Mianyang 26 / Yumai 47; (C) Chao index of OTU level of inoculated Mianyang 26 / Yumai 47; (D) Shannon index of OTU level of inoculated A-45; (E) Ace index of OTU level of inoculated A-45; (F) Chao index of OTU level of inoculated A-45. The difference test method between groups was Student's T test, and P ≤ 0.05 was marked as *. R, resistant variety inoculated with *T. controversa*; S, susceptible variety inoculated with *T. controversa*; 26, wheat hybrid variety Mianyang 26 / Yumai 47; 45, wheat variety A-45.

**Supplementary Table 1.** OTU species classification statistics table. T, soil; R, root; E, ear; SB, stem base; FS, first stem under the ear. R, resistant variety inoculated with *T. controversa*; S, susceptible variety inoculated with *T. controversa*; CK, wheat not inoculated with *T. controversa*; 26, wheat hybrid variety Mianyang 26 / Yumai 47; 49, wheat hybrid variety Yumai 49 * 4 / Lankao dwarf 8; 44, wheat variety A-44; 45, wheat variety A-45.

**Supplementary Table 2**. Characterization of the microbial richness and diversity index of soil fungi. Treatment values within a row followed by different letters are significantly differences at P ≤ 0.05 levels according to Student's t test.T, soil. R, resistant variety inoculated with *T. controversa*; S, susceptible variety inoculated with *T. controversa*; CK, wheat not inoculated with *T. controversa*; 26, wheat hybrid variety Mianyang 26 / Yumai 47; 49, wheat hybrid variety Yumai 49 * 4 / Lankao dwarf 8; 44, wheat variety A-44; 45, wheat variety A-45.

**Supplementary Table 3.** Characterization of the microbial richness and diversity index of soil bacteria. Treatment values within a row followed by different letters are significantly differences at P ≤ 0.05 levels according to Student's t test.T, soil. R, resistant variety inoculated with *T. controversa*; S, susceptible variety inoculated with *T. controversa*; CK, wheat not inoculated with T. controversa; 26, wheat hybrid variety Mianyang 26 / Yumai 47; 49, wheat hybrid variety Yumai 49 * 4 / Lankao dwarf 8; 44, wheat variety A-44; 45, wheat variety A-45.

**Supplementary Table 4.** Characterization of the microbial richness and diversity index of bacteria in root. Treatment values within a row followed by different letters are significantly differences at P ≤ 0.05 levels according to Student's t test. R (front): root. R (back), resistant variety inoculated with *T. controversa*; S, susceptible variety inoculated with *T. controversa*; CK, wheat not inoculated with T. controversa; 26, wheat hybrid variety Mianyang 26 / Yumai 47; 49, wheat hybrid variety Yumai 49 * 4 / Lankao dwarf 8; 44, wheat variety A-44; 45, wheat variety A-45.

**Supplementary Table 5.** Characterization of the microbial richness and diversity index of bacteria in the ear. Treatment values within a row followed by different letters are significantly differences at P ≤ 0.05 levels according to Student's t test.E, ear. R, resistant variety inoculated with *T. controversa*; S, susceptible variety inoculated with *T. controversa*; CK, wheat not inoculated with *T. controversa*; 26, wheat hybrid variety Mianyang 26 / Yumai 47; 45, wheat variety A-45.

**Supplementary Table 6.** Characterization of the microbial richness and diversity index of bacteria in the first stem. Treatment values within a row followed by different letters are significantly differences at P ≤ 0.05 levels according to Student's t test.FS, first stem under the ear. R, resistant variety inoculated with *T. controversa*; S, susceptible variety inoculated with *T. controversa*; CK, wheat not inoculated with *T. controversa*; 26, wheat hybrid variety Mianyang 26 / Yumai 47; 45, wheat variety A-45.

**Supplementary Table 7.** Characterization of the microbial richness and diversity index of bacteria in stem base. Treatment values within a row followed by different letters are significantly differences at P ≤ 0.05 levels according to Student's t test.SB, stem base. R, resistant variety inoculated with *T. controversa*; S, susceptible variety inoculated with *T. controversa*; CK, wheat not inoculated with *T. controversa*; 26, wheat hybrid variety Mianyang 26 / Yumai 47; 45, wheat variety A-45.

**Supplementary Table 8.** Topological properties of bacterial co-occurring networks associated with inoculated and uninoculated wheat groups.

**Supplementary Table 9.** Topological properties of fungal co-occurring networks associated with inoculated and uninoculated wheat groups.
