# Supplementary figures and images for: Characterization of the microbial communities in wheat tissues and rhizosphere soil caused by dwarf bunt of wheat
Source: Sci Rep. 2021 Mar 11;11:5773. doi: 10.1038/s41598-021-85281-8 (PMC7952392; doi:10.1038/s41598-021-85281-8)

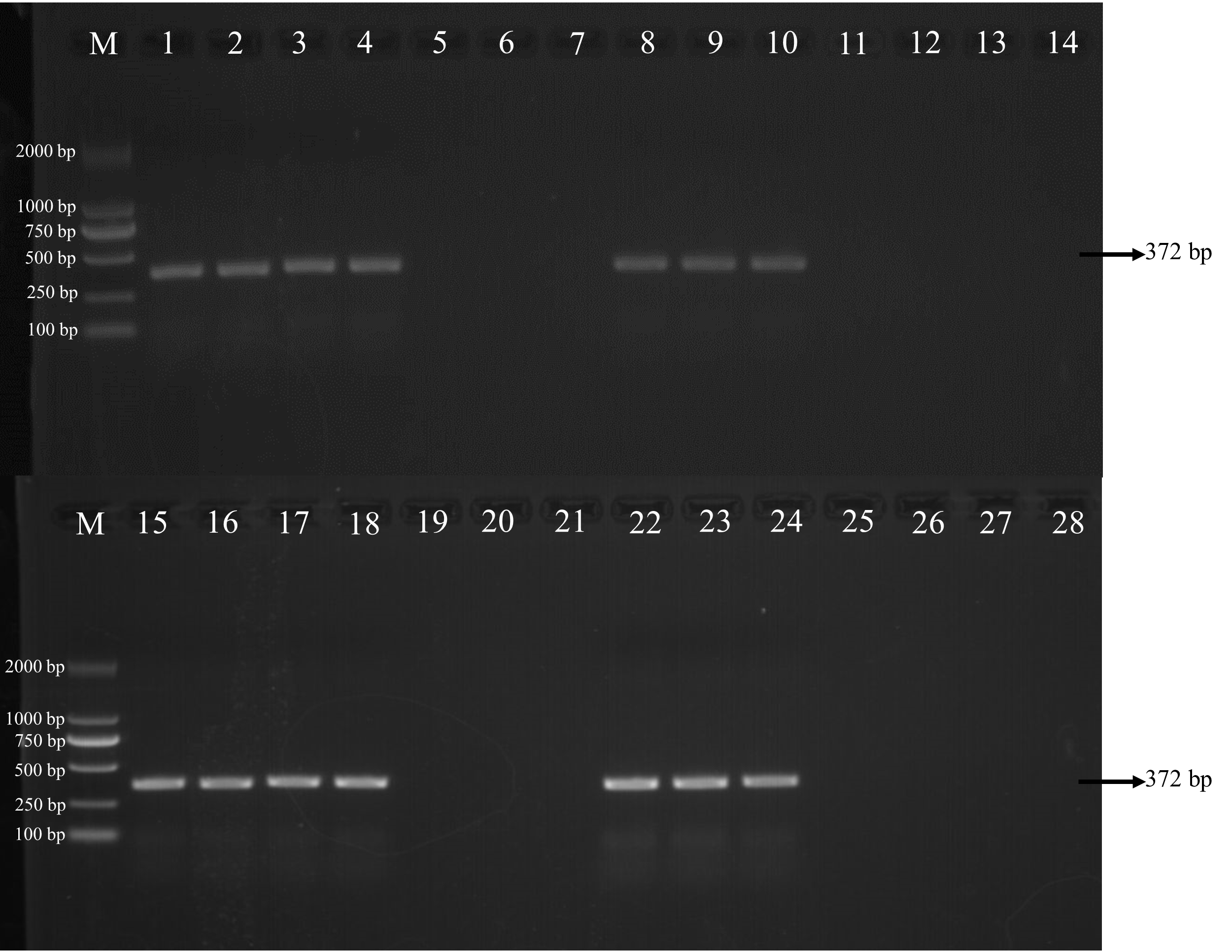

Supplement: Supplementary file 2 — Supplementary Figure 1. [file 41598_2021_85281_MOESM2_ESM.tif]

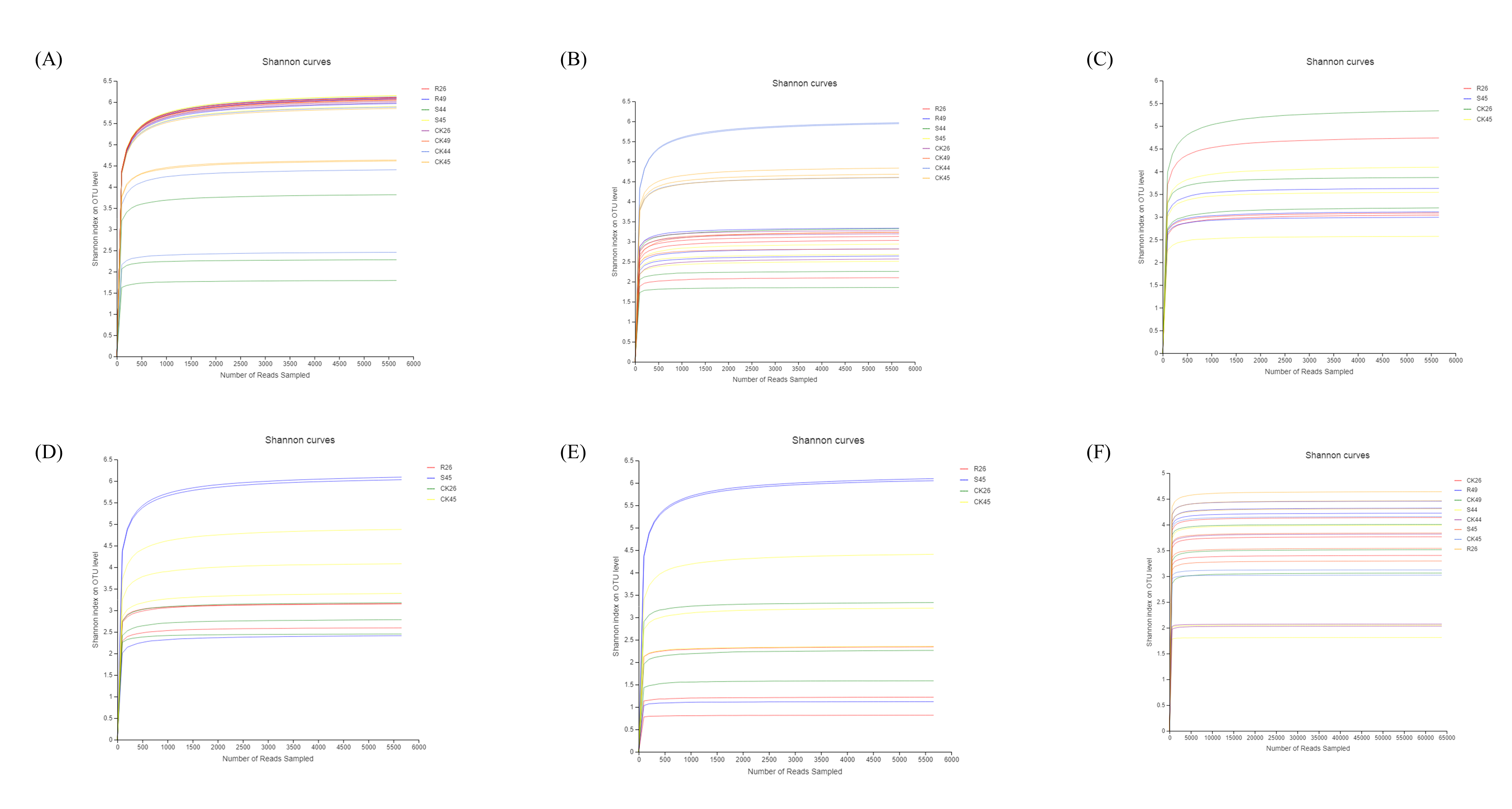

Supplement: Supplementary file 3 — Supplementary Figure 2. [file 41598_2021_85281_MOESM3_ESM.tif]

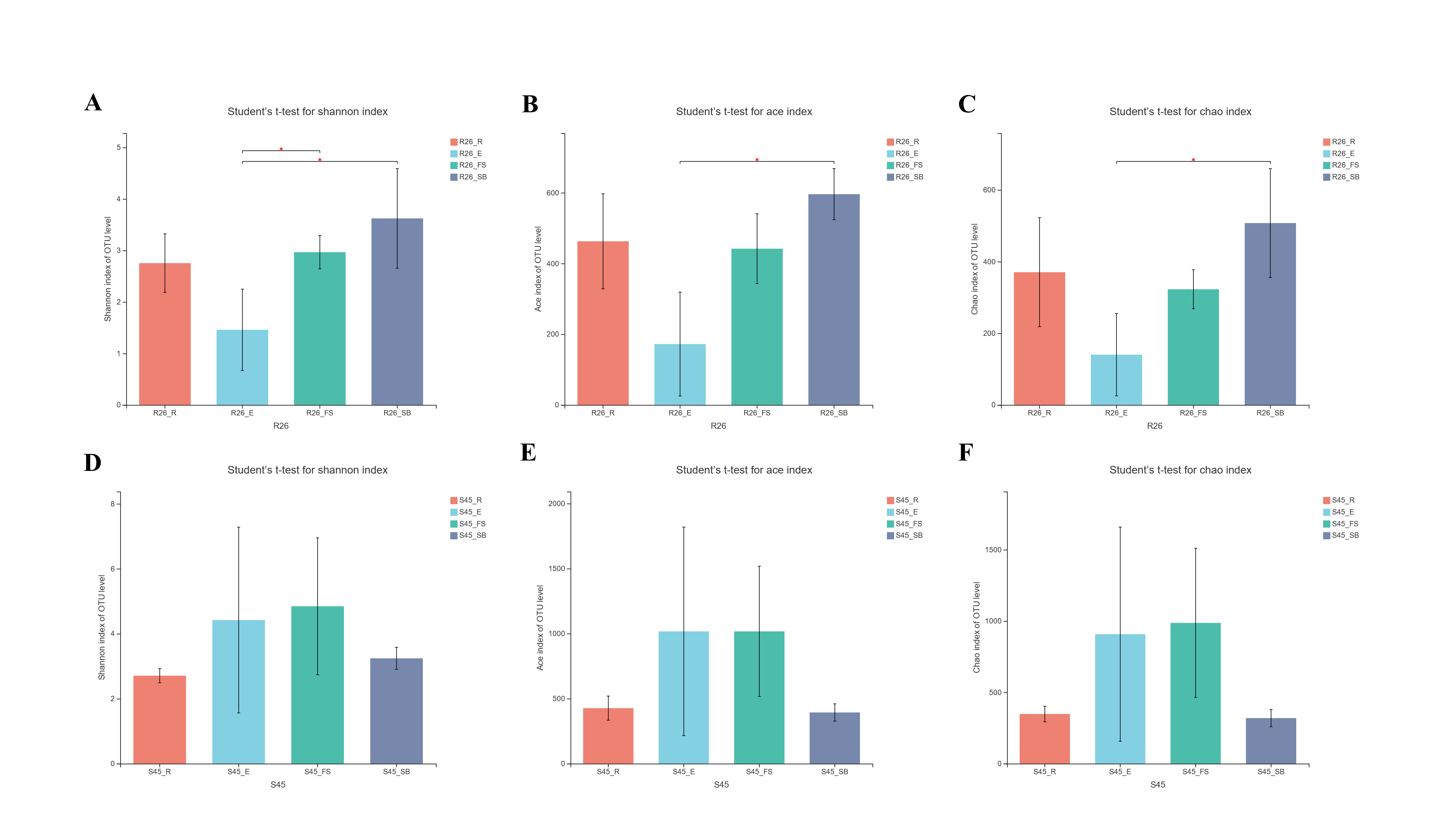

Supplement: Supplementary file 4 — Supplementary Figure 2. [file 41598_2021_85281_MOESM4_ESM.tif]
